# Characteristic of the complete chloroplast genome of *Symphoricarpos sinensis*, an important ornamental plant endemic to China

**DOI:** 10.1080/23802359.2020.1807423

**Published:** 2020-08-14

**Authors:** Yi Zhang, Qing Yuan Zhang, Hang Ran, Yanan Cao

**Affiliations:** College of Plant Protection, Henan Agricultural University, Zhengzhou, China

**Keywords:** Complete chloroplast genome, Caprifoliaceae, *Symphoricarpos*

## Abstract

*Symphoricarpos sinensis* is an important ornamental plant in China. Due to the lack of efficient molecular markers, although recent studies supported that *Symphoricarpos* was phylogenetically closely related to *Lonicera*, *Triosteum*, *Heptacodium*, and *Leycesteria*, relationships among these taxa remained uncertain. To examine the phylogenetic position of *Symphoricarpos* within Caprifoliaceae, we characterized the complete chloroplast (cp) genome of *S. sinensis*. The results showed that *S. sinensis* shared a typical quadripartite structure as with most angiosperms. It was 155,738 bp in length, including two inverted repeat (IR) regions (24,010 bp), a small single-copy (SSC) region (18,942 bp) and a large single-copy (LSC) region (88,776 bp). Phylogenetic analysis demonstrated that Caprifoliaceae and Adoxaceae formed two distinct monophyletic clades, and *S. sinensis* was closely related to *lonicera* and *Triosteum*, albeit with low support. The whole cp genome of *S. sinensis* will be useful resources for future studies on phylogeny and conservation in *Symphoricarpos.*

*Symphoricarpos* (Caprifoliaceae) is an upright shrub distributed in North America, Mexico and China. Based on recent molecular phylogenetic studies, *Symphoricarpos* was found to be closely related to *Lonicera*, *Triosteum*, *Heptacodium*, and *Leycesteria*, however, phylogenetic relationships among these taxa remained controversial (Xiang et al. 2020). In recent years, the whole cp genomes have become valuable resources for molecular phylogeny and species identification due to highly conservative structure and low evolutionary rate (Liu et al. 2017; Liang et al. 2018). In this study, we reported the complete cp genome of *S. sinensis*, which will be helpful for further studies on phylogeny in *Symphoricarpos*.

Fresh materials were collected in Zhongshan Botanical Garden (N 32°03'16.85″, E 118°49′52.06″) and the voucher specimen was stored in Henan Agricultural University Herbarium (CYN2019052202). The whole cp genome of *S. sinensis* was sequenced on an Illumina Hiseq2500 platform at Suzhou Jinweizhi Biotechnology Institute. Firstly, the filtration and assembly of chloroplast genomes was completed at CLC Genomics Workbench (http://www.clcbio.com). Then, the cp genome of *S. sinensis* was annotated in PGA (http://github.com/quxiaojian/PGA) using the *Lonicera japonica* (NC_026839.1), *Heptacodium miconioides* (NC_042739) and *Triosteum pinnatifidum* (NC_037952) as references. The start/stop codons and intron/exon boundaries of genes were subsequently manually modified based on the reference sequences. Next, tRNAscan-SE v1.21 (Schattner et al. 2005) was further used to examine the tRNA boundaries with default settings, and then the physical map of the cp genome was drawn using the online program Organellar Genome DRAW (Lohse et al. 2013). Finally, to infer the phylogenetic position of *Symphoricarpos* within Caprifoliaceae, 15 species of *Lonicera* and 8 species representing 8 genera of Caprifoliaceae were selected to construct the phylogenetic tree using PhyML v3.0 (Guindon et al. 2010). Six species (i.e. *Adoxa moschatellina*, *Tetradoxa omeiensis*, *Sinadoxa corydalifolia*, *Viburnum utile*, *Viburnum betulifolium* and *Sambucus williamsii*) were selected as outgroups.

The cp genome of *S. sinensis* (GenBank accession number: MT507606) harbored a typical quadripartite structure with a full length of 155,738 bp and 38.4% GC content, comprising two inverted repeat (IR) regions (24,010 bp), a LSC region (88,776 bp) and a SSC region (18,942 bp). It encoded 131 functional genes, including 84 protein-coding genes, 39 transfer RNA (tRNA) genes, 8 ribosomal RNA (rRNA) genes. Of those protein-coding genes, 9 contained one intron and 4 contained two introns. It was worth noting that 16 genes were completely duplicated in the IR regions. The overall structure, gene content and arrangement of the cp genome of *S. sinensis* was quite similar to those of other reported Caprifoliaceae species (He et al. 2017; Wang et al. 2020).

The phylogenetic analysis (Figure 1) recovered two well-supported monophyletic clades (both 100% bootstrap values) representing Caprifoliaceae and Adoxaceae, respectively, and showed that *S. sinensis* was closely related to *lonicera* and *Triosteum* within Caprifoliaceae, albeit with low bootstrap support (38%). These results were largely consistent with previous studies (Bell 2010; Xiang et al. 2020).

**Figure 1.**
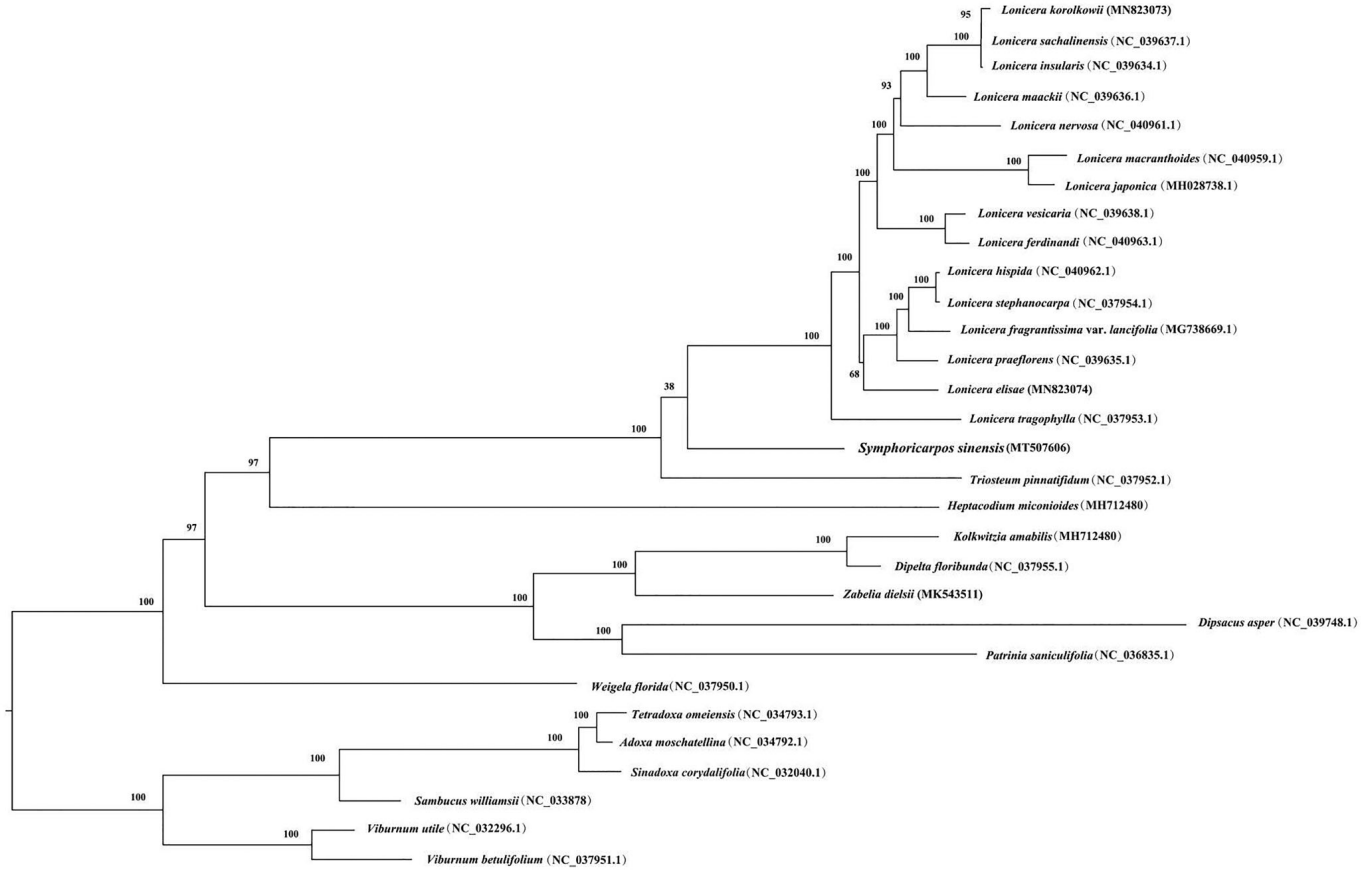
ML phylogenetic tree inferred from 30 complete chloroplast genome sequences of Dipsacales. Numbers above each node indicate bootstrap values.

## Data Availability

The data that support the findings of this study are openly available in NCBI at https://www.ncbi.nlm.nih.gov/WebSub/?form=history&tool=genbank, reference number [MT507606].
